# Genetic Variant of* Kalirin* Gene Is Associated with Ischemic Stroke in a Chinese Han Population

**DOI:** 10.1155/2017/6594271

**Published:** 2017-06-19

**Authors:** Hong Li, Shasha Yu, Rui Wang, Zhaoqing Sun, Xinghu Zhou, Liqiang Zheng, Zhihua Yin, Xingang Zhang, Yingxian Sun

**Affiliations:** ^1^Department of Cardiology, Shengjing Hospital of China Medical University, 36 Sanhao Street, Shenyang 110004, China; ^2^Department of Cardiology, The First Hospital of China Medical University, 155 North Nanjing Street, Shenyang 110001, China; ^3^Department of Clinical Epidemiology, Library, Shengjing Hospital of China Medical University, 36 Sanhao Street, Shenyang 110004, China; ^4^Epidemiology Department of China Medical University, No. 77 Puhe Road, Shenyang 110000, China

## Abstract

**Introduction:**

Ischemic stroke is a complex disorder resulting from the interplay of genetic and environmental factors. Previous studies showed that kalirin gene variations were associated with cardiovascular disease. However, the association between this gene and ischemic stroke was unknown. We performed this study to confirm if kalirin gene variation was associated with ischemic stroke.

**Methods:**

We enrolled 385 ischemic stroke patients and 362 controls from China. Three SNPs of kalirin gene were genotyped by means of ligase detection reaction-PCR method. Data was processed with SPSS and SHEsis platform.

**Results:**

SNP rs7620580 (dominant model: OR = 1.590,* p* = 0.002 and adjusted OR = 1.662,* p* = 0.014; additive model: OR = 1.490,* p* = 0.002 and adjusted OR = 1.636,* p* = 0.005; recessive model: OR = 2.686,* p* = 0.039) and SNP rs1708303 (dominant model: OR = 1.523,* p *= 0.007 and adjusted OR = 1.604,* p* = 0.028; additive model: OR = 1.438,* p* = 0.01 and adjusted OR = 1.476,* p* = 0.039) were associated with ischemic stroke. The GG genotype and G allele of SNP rs7620580 were associated with a risk for ischemic stroke with an adjusted OR of 3.195 and an OR of 1.446, respectively. Haplotype analysis revealed that A–T–G,G-T-A, and A-T-A haplotypes were associated with ischemic stroke.

**Conclusions:**

Our results provide evidence that kalirin gene variations were associated with ischemic stroke in the Chinese Han population.

## 1. Introduction

Cerebrovascular diseases and cardiovascular diseases are among the top three leading causes of death and disability worldwide [1, 2]. The mortality rate due to stroke is as high as 276.9/100,000 each year in China [3], which means nearly 400,000 people die of stroke every year. Ischemic stroke (IS) is a complex disorder resulting from the interplay of genetic and environmental factors (such as age, smoking, hypertension, cardiac arrhythmias, and diabetes mellitus) [4]. It has been shown that genetic factors increase the risk of stroke. The risk rate of a person with a family history of stroke is up to 30% [5]. Compared with dizygotic twins, the rates of death and hospitalization due to stroke were increased for monozygotic twins [6].

Present prevention and treatment of stroke are involved in the control of risk factors [7] and revascularization of vascular stenosis. Medical therapy was often effective for preventing stroke. However, some patients may still experience recurrent ischemic stroke in spite of receiving aggressive medical treatment [8]. With the rapid advance of precise medicine, it is of great importance to find and validate biomarkers for the diagnosis, treatment, and prognosis of stroke.

The protein encoded for by the* kalirin gene (KALRN)* is a guanine nucleotide exchange factor (GEF), which has numerous functions, such as neuron morphogenesis [9, 10], modifies synphilin-1 aggregate transport [11], and promotes smooth muscle cell (SMC) migration and proliferation [12]. Studies found that* KALRN* gene variations were associated with CHD [13–16]. As an atherosclerotic disease, stroke may share some genes with coronary heart disease (CHD). A possible association between* KALRN* gene polymorphisms and IS among distinct population remains unknown. Thus, the aim of the present study was to investigate if* KALRN* SNPs were associated with ischemic stroke among Han Chinese.

## 2. Materials and Methods

### 2.1. Subjects

A cohort of 747 individuals, including 385 ischemic stroke patients and 362 hypertensive controls without stroke, was included in the present study. The ischemic stroke group was composed of 297 patients found during the epidemiological survey of Fuxin rural areas (Liaoning province, China) [17] and 88 ischemic stroke patients from the First Hospital of China Medical University. The study subjects were unrelated to one another, were all Han Chinese, and were older than 35 years of age. Subjects were excluded if they had a history of hemorrhagic stroke, tumor, trauma, myocardial infarction, or atrial arrhythmia. All IS cases were confirmed to be atherosclerotic ischemic stroke by brain imaging using computed tomography (CT) scanning and/or magnetic resonance imaging (MRI). A diagnosis of IS was confirmed by 2 neurologists.

The control group included unrelated individuals without any history or symptoms of cerebrovascular disease and were matched with the IS patients for residency area, ethnic origin, and gender. Brain imaging was not used for controls. As stroke is a late-onset disease, we selected older people as controls to minimize the chances for misclassification as “stroke-free.”

All participants were given a questionnaire that included demographic variables (age, gender, and nationality). Histories of conventional risk factors (hypertension, diabetes mellitus, smoking, and alcohol drinking) were also recorded. Blood pressure (BP) was measured using a standardized automated electronic sphygmomanometer (Omron, Dalian, China). Biochemical analyses were done using an automated enzymatic procedure (AU640, Olympus, Japan) in our central laboratory. After providing informed consent, 5 ml venous blood was obtained from each subject and kept at –20°C until analyzed.

The work has been carried out in accordance with the Code of Ethics of the World Medical Association (Declaration of Helsinki) for experiments involving human subjects. Our procedures were approved by the Ethics Committee of China Medical University, and written informed consent was obtained from all participants.

### 2.2. SNP Selection and Genotyping

Genomic DNA was isolated from EDTA anticoagulated whole blood of subjects in the laboratory of Shengjing Hospital of China Medical University using blood genomic DNA extraction kits according to the manufacturer's protocol (TIANamp Genomic DNA kit, Tiangen Biochemical Technology, Beijing, China) and kept at −20°C until analyzed.

The three selected SNPs for the present study were tag-SNPs selected from HapMap databases: rs7620580 (TTTGCTTGGTAGTGGTTGCATGTGC[A/G]TGTGTGCATGTGTGGACTGTCTGCT, Chromosome: 3: 124326456; Functional Consequence: intron variant), rs2289843 (AGGTGACATACATCTGAAATTTGTC[A/T]GCCTAAAAAGAACAAAGATAATAAT, Chromosome: 3:  124477247; Functional Consequence: synonymous codon) and rs1708303 (AGAATGGAGGCAAGTCCGAGTCCGT[A/G]GCCAACCTGCAGGCCCAGCCCTCCC, Chromosome: 3: 124632469; Functional Consequence: synonymous codon). The SNPs were to cover the genes of interest with an* r*^2^ threshold of 0.8 and minor allele frequency (MAF) greater than 0.1. Genotyping was done using the ligase detection reaction method (LDR-PCR) [18–20]. Primers were designed with primer 5 and probes were designed by Shanghai Generay Biotech (http://www.generay.com.cn/) (Table 1). Allele and genotype frequencies were determined by analyzing the raw data from ABI 3730XL with Peak Scanner Software v1.0 (ABI). About 10% randomly selected samples were genotyped repeatedly to ensure that the results were 100% concordant.

### 2.3. Statistical Analysis

Statistical analyses were done using SPSS (v 13.0) and the SHEsis analysis platform (http://analysis.bio-x.cn/myAnalysis.php). Data are presented as mean ± standard deviation (SD) with normal distribution by using the *t*-test. Count data are presented as number of cases and percentage. Pearson Chi-square (*X*^2^) tests or Fisher's exact test were used to compare the frequencies of demographic variables and to assess differences in SNP genotypes and alleles between cases and controls. Hardy-Weinberg equilibrium (HWE) was assessed using *X*^2^ tests. Odds ratios (OR) with 95% confidence intervals (CI) were determined using unconditional logistic regression analysis. Multivariate analysis was evaluated by logistic regression models to test the association of gene variation and ischemic stroke risk after adjusting the confounding risk factors. The SHEsis analysis platform was used to calculate linkage disequilibrium indices (*D*′ and* r*^2^) and inferred haplotype frequencies [21, 22].

## 3. Results

### 3.1. Subject Characteristics

The demographic and clinical characteristics of the subjects were shown in Table 2. There were no significant differences between cases and controls for gender, heart rate, or total cholesterol levels. Because we chose cases that were younger than controls, their mean ages were different (62.30 ± 9.75 versus 69.01 ± 7.32 years). As expected, the prevalence of the most common risk factors for atherosclerosis was significantly different between cases and controls: body mass index (BMI) (*p* = 0.006), drinking (*p* = 0.006), smoking (*p* = 0.039), triglyceride (TG) (*p* = 0.006), low density lipid cholesterol (LDL-C) (*p* < 0.001), high density lipid cholesterol (HDL-C) (*p* < 0.001), and fasting plasma glucose (FPG) (*p* = 0.035).

### 3.2. Association between SNPs and Ischemic Stroke Risk

The genotype distributions and allelic frequencies for the 3 SNPs that we selected among cases and controls were shown in Tables 3 and 4. All allele distributions of the studied SNPs were in HWE.

For a dominant model, analyses were done by combining the heterozygous variant genotype with the homozygous variant genotype. For a recessive model, analyses were done by combining the heterozygous variant genotype with the wild type homozygous genotype. As shown in Table 3, SNP rs7620580 (dominant model: OR = 1.590 (95% CI 1.187–2.130), *p* = 0.002; additive model: OR = 1.490 (95% CI 1.157–1.919), *p* = 0.002) and SNP rs1708303 (dominant model: OR = 1.523 (95% CI 1.119–2.073), *p* = 0.007; additive model: OR = 1.438 (95% CI 1.092–1.892), *p* = 0.01) were significantly associated with ischemic stroke in this population. The GG genotype and G allele of SNP rs7620580 were associated with a risk for ischemic stroke with an adjusted OR of 3.195 (95% CI 1.228–8.312, *p* = 0.017) and OR of 1.446 (95% CI 1.136–1.841, *p* = 0.003), respectively (Tables 3 and 4). However, there were no significant differences in the allele and genotype distributions for SNP rs2289843 between cases and controls in total subjects.

Because some conventional confounding risk factors may also contribute to the development of stroke, we analyzed the associations between these SNPs and IS after adjusting for these factors. Multivariate logistic regression analysis showed that, after adjusting for age, systolic pressure, diastolic pressure, BMI, smoking, drinking, TG, HDL-C, LDL-C, and FPG, SNP rs7620580 (dominant model: OR = 1.662 (95% CI 1.110–2.487), *p* = 0.014; additive model: OR = 1.636 (95% CI 1.163–2.300), *p* = 0.005; recessive model: OR = 2.686 (95% CI 1.049–6.879), *p* = 0.039) and SNP rs1708303 (dominant model: OR = 1.604 (95% CI 1.051–2.447), *p* = 0.028; additive model: OR = 1.476 (95% CI 1.020–2.135), *p* = 0.039) still showed associations with ischemic stroke.

We found an interaction between the gene variation and environment (Table 5). So we also studied the association of the* KALRN* SNPs and IS under different environmental exposures, such as gender and overweight (BMI ≥ 25 kg/m^2^) (Table 6). Genotypic association of SNP rs7620580 was also revealed in men before and after adjustment of covariates in the additive and dominant models. But in women, significant difference was present only after adjustment of covariates (additive model: OR = 1.804 (95% CI 1.032–3.152), *p* = 0.038, dominant model OR = 2.007 (95% CI 1.062–3.793), *p* = 0.032). The association of SNP rs1708303 and IS was present in women in the crude additive and dominant model (OR = 1.566 (95% CI 1.045–2.345), *p* = 0.030, and OR = 1.665 (95% CI 1.046–2.652), *p* = 0.032, resp.) but was lost after adjustment. No association of SNP rs1708303 and IS was found in men (*p* > 0.05). SNP rs2289843 genotypes were associated with IS only in women after adjustment for covariates in recessive and additive model (OR = 2.186 (95% CI 1.035–4.620), *p* = 0.040, and OR = 1.658 (95% CI 1.059–2.595), *p* = 0.027, resp.). As shown in Table 6, the genotype distributions of rs7620580 and rs1708303 were also different between overweight subjects and nonoverweight subjects (BMI < 25 kg/m^2^). No genotypic association between SNP rs2289843 and IS was revealed in subjects with different BMI (*p* > 0.05).

Thus, we concluded that rs7620580, rs1708303, and rs2289843 polymorphisms may have been associated with a risk for the development of IS among Han Chinese (Table 3).

### 3.3. Haplotype Analysis

**Figure 1:**
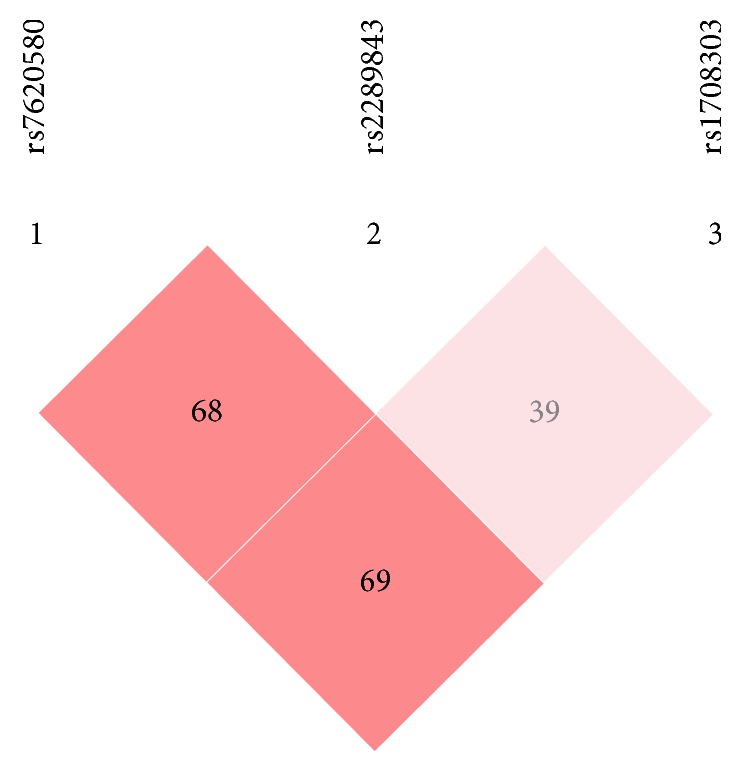
*D*′ of the 3 SNPs: it showed that they were in linkage disequilibrium.

**Figure 2:**
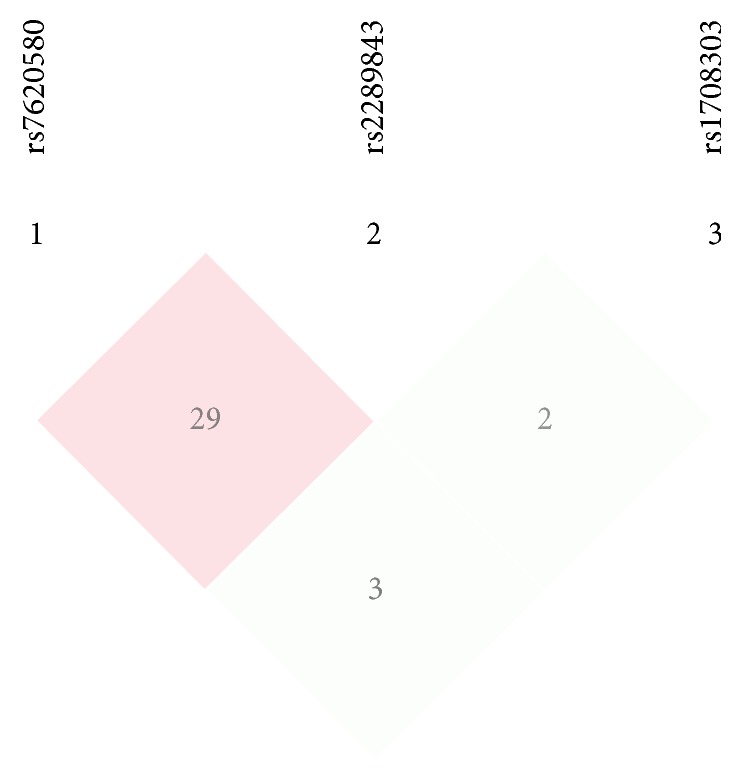
*r* ^2^ of the 3 SNPs: it showed that they were in linkage disequilibrium.

We also analyzed haplotypes using SHEsis program platform. Haplotypes were constructed in* KALRN *based on the three SNPs (rs7620580, rs2289843, and rs1708303). These 3 SNPs were in linkage disequilibrium in this study population (Figures 1 and 2). Of 8 possible haplotypes, only 6 had frequencies of >0.03 among both cases and controls and were included in our haplotype analysis (Table 7). The frequencies of the A–T–G and G-T-A haplotypes in cases were significantly higher than that in controls (12.0% versus 6.0%, *p* < 0.001 and 11.0% versus 6.9%, *p* = 0.006, resp.). The frequencies of A-T-A haplotype in cases were significantly lower than those in controls (26.2% versus 38.3%,* p* < 0.001).

## 4. Discussion

In this study, we investigated possible associations between 3 polymorphisms of the* KALRN* gene and ischemic stroke in a northern Chinese Han population. We found that SNP rs7620580, rs2289843, and rs1708303 were associated with IS and revealed that the G allele of SNP rs7620580 and G allele of SNP rs1708303 were risk factors for IS. To the best of our knowledge, this is the first study to demonstrate that these three SNPs were associated with the occurrence of ischemic stroke in Chinese population.

Age and hypertension are independent risk factors for stroke. As with other atherosclerotic diseases, stroke is a late-onset disease. Susceptibility genes can influence an early-onset of this disease when environmental factors have not had sufficient time to modify the phenotype. Several studies of the* MTHFR* and* apolipoprotein E* genes [23],* PDE4D* genes [24], and variations on chromosome 9p21 [25] have shown age-dependent effects. Thus, we selected place of residence and sex-matched, older, and higher blood pressure patients without stroke as controls to minimize the chances of misclassification as “stroke-free” and to exclude the impact of hypertension in the control group.

The protein encoded for by the* KALRN* gene is a complex protein with multiple catalytic, protein-protein, and protein-lipid interaction domains. It is involved in neuron morphogenesis [9, 10] and granule maturation [26, 27] and modifies synphilin-1 aggregate transport and formation [11]. Kalirin 7 is the most abundant isoform of kalirin in the adult brain that is nearly exclusively localized to the postsynaptic density (PSD) [28, 29] and plays an essential role in dendritic spine formation and function [30–33]. Kalirin 7 is also involved in ischemic signal transduction [34]. Nitric oxide (NO) acts as a neurotransmitter. Kalirin inhibits inducible nitric-oxide synthase (iNOS) activity [35] and promotes SMC migration and proliferation both in vitro and in vivo [12]. Kalirin's RhoGEF activity could plausibly augment atherogenesis by enhancing vascular SMC proliferation and migration [12, 36–38] and endothelial dysfunction [39].

The* KALRN* gene has been linked to schizophrenia [40, 41] and adult attention deficit-hyperactivity disorder [42]. Recent studies found that* KALRN* gene variations were associated with atherosclerotic diseases, such as coronary heart disease [13, 15] and stroke [43, 44], whereas Olsson et al. [45] found no linkage. These differences may have been due to the different ethnic groups that were studied. Different IS risk factors may also affect the results of association study. As we have shown in Table 6, the associations were presented in man or thinner subjects, while they disappeared in woman or overweight ones. So the difference of our study and Olsson's may have been also due to different risk factors of the study subjects. Besides genetic and environmental backgrounds, differences in sample size, population admixture, and different selection criteria may explain the discrepancy, too.

The studied SNPs rs1708303 and rs2289843 were located in exons. We found that an intronic locus SNP rs7620580 was associated with IS, too. Perhaps polymorphisms in the intron region are in linkage disequilibrium with unidentified variants in regulatory elements, which affect gene transcription rates or gene expression efficiency. It is possible that an intron SNP interferes with nucleotide splicing or the formation of different spliceosomes, which might change gene structure and influence protein folding.

There were several limitations for our study. First, our subjects were selected from the rural elderly patients in Northern China during an epidemiological investigation; the conclusion may not represent populations of other genetic background. Further large-scale, randomized, long-term studies from different regions and ethnic or social background will be needed to confirm our present findings. Second, one of our selected SNPs (rs 7620580) was located in introns, the function of which was not clear. Third, because stroke is a disease that might involve multiple SNPs among multiple genes, a comprehensive analysis of the interactions between candidate genes will be more powerful than a single-locus analysis. Finally, functional studies are needed to confirm our findings from this study.

## 5. Conclusions

Our results provide evidence that* KALRN* gene variations were associated with ischemic stroke in the Chinese Han population.

## Figures and Tables

**Table 1:** Primers and probes.

SNP	Primers	Probes
rs7620580	F: GGTAACTCTTGGTGACTTTGC	TA: TTTTTTTTTTGCTTGGTAGTGGTTGCATGTGCA
	R: CATTCACCCCTTTGATCTGTG	TG: TTTTTTTTTTTTTGCTTGGTAGTGGTTGCATGTGCG
		TR: -P-TGTGTGCATGTGTGGACTGTCTGCTTTTTTT-FAM-
rs2289843	F: CATTATGCCAAAGCAGAGAAG	TA: GGTGACATACATCTGAAATTTGTCA
	R: TGGTGGACAGAAGGGAACAAC	TT: TTTGGTGACATACATCTGAAATTTGTCT
		TR: -P-GCCTAAAAAGAACAAAGATAATAAT-FAM-
rs1708303	F: TTCTCTGGACTGGGGTCTACA	TA: TTTTGAATGGAGGCAAGTCCGAGTCCGTA
	R: ACAGGACTCGTCAGCCACTTC	TG: TTTTTTTGAATGGAGGCAAGTCCGAGTCCGTG
		TR: -P-GCCAACCTGCAGGCCCAGCCCTCCCTTT-FAM-

**Table 2:** Study subjects' characteristics.

	Cases	Controls	*p*
Age (years)	62.30 ± 9.75	69.01 ± 7.32	<0.001
Gender (male/female, *n*)	219/166	209/153	0.814
Heart rate (bpm)	76.44 ± 9.66	76.29 ± 12.19	0.849
SBP (mmHg)	164.27 ± 23.47	189.88 ± 16.38	<0.001
DBP (mmHg)	95.70 ± 14.47	102.32 ± 13.00	<0.001
BMI (kg/m^2^)	24.10 ± 3.71	23.29 ± 4.16	0.006
Drinking (%)	95 (24.7%)	123 (34.0%)	0.006
Smoking (%)	156 (40.5%)	174 (48.1%)	0.039
CHOL (mmol/L)	5.35 ± 1.05	5.40 ± 1.03	0.499
TG (mmol/L)	1.89 ± 1.47	1.62 ± 1.17	0.006
LDL-C (mmol/L)	3.08 ± 0.75	2.85 ± 0.71	<0.001
HDL-C (mmol/L)	1.35 ± 0.33	1.48 ± 0.32	<0.001
FPG (mmol/L)	6.25 ± 2.22	5.93 ± 1.96	0.035

Note: *n*, number; SBP, systolic pressure; DBP, diastolic pressure; BMI, body mass index; TG, triglyceride; CHOL, total cholesterol; HDL-C, high density lipid cholesterol; LDL-C, low density lipid cholesterol; FPG, fasting plasma glucose.

**Table 3:** Genotype distributions and detailed association results.

Genotype	IS (*n*)	Control (*n*)	OR (95% CI)	*p*	OR (95% CI)^*∗*^	*p* ^*∗*^
rs7620580						
AA	199	228	1.000		1.000	
AG	166	122	**1.559 (1.153–2.107)**	**0.004 **	**1.534 (1.011–2.328)**	**0.045 **
GG	20	12	1.910 (0.911–4.004)	0.087	**3.195 (1.228–8.312)**	**0.017 **
Recessive			1.598 (0.770–3.318)	0.208	**2.686 (1.049–6.879)**	**0.039 **
Additive			**1.490 (1.157–1.919)**	**0.002 **	**1.636 (1.163–2.300)**	**0.005 **
Dominant			**1.590 (1.187–2.130)**	**0.002 **	**1.662 (1.110–2.487)**	**0.014 **
rs2289843						
TT	101	107	1.000		1.000	
TA	195	181	1.141 (0.813–1.602)	0.445	1.291 (0.809–2.059)	0.285
AA	89	74	1.274 (0.845–1.922)	0.248	1.402 (0.807–2.434)	0.231
Recessive			1.170 (0.826–1.658)	0.377	1.197 (0.748–1.914)	0.453
Additive			1.130 (0.920–1.386)	0.243	1.189 (0.903–1.566)	0.216
Dominant			1.180 (0.857–1.625)	0.311	1.326 (0.856–2.053)	0.206
rs1708303						
AA	241	260	1.000		1.000	
AG	132	94	**1.515 (1.103–2.081)**	**0.010 **	**1.610 (1.040–2.490)**	**0.033 **
GG	12	8	1.618 (0.650–4.027)	0.301	1.550 (0.484–4.969)	0.461
Recessive			1.424 (0.575–3.524)	0.445	1.338 (0.421–4.250)	0.622
Additive			**1.438 (1.092–1.892)**	**0.010 **	**1.476 (1.020–2.135)**	**0.039 **
Dominant			**1.523 (1.119–2.073)**	**0.007 **	**1.604 (1.051–2.447)**	**0.028 **

^*∗*^adjusted for age, SBP, DBP, FPG, TG, HDL, LDL, smoking, drinking, and BMI.

**Table 4:** SNP allele frequencies in cases and controls.

Allele	Cases (*n*)	Controls (*n*)	OR (95% CI)	*p*	HWE P in controls
rs7620580					0.37
A allele	564	578	1	—	
G allele	206	146	**1.446 (1.136–1.841)**	**0.003**	
rs2289843					0.87
T allele	397	395	1	—	
A allele	373	329	1.128 (0.920–1.382)	0.246	
rs1708303					0.88
A allele	614	614	1	—	
G allele	156	110	**1.418 (1.084–1.855)**	**0.011**	

**Table 5:** Interaction of three SNPs and environmental risk factors on IS.

Genotype	Gender	IS (*n*)	Control (*n*)	OR (95% CI)	*p*

rs7620580					
AA	M	109	133	1	
	F	90	95	1.156 (0.788–1.697)	0.459
AA + AG	M	110	76	**1.766 (1.200–2.600)**	**0.004**
	F	76	58	0.783 (0.434–1.415)	0.418
rs2289843					
TT + TA	M	166	158	1	
	F	130	130	1.171 (0.822–1.668)	0.383
AA	M	53	51	0.954 (0.591–1.542)	0.849
	F	36	23	1.736 (0.799–3.770)	0.163
rs1708303					
AA	M	143	152	1	
	F	98	108	1.212 (0.823–1.784)	0.331
AA + AG	M	76	57	1.444 (0.928–2.247)	0.104
	F	68	45	1.182 (0.607–2.303)	0.623

Genotype	Overweight	IS (*n*)	Control (*n*)	OR (95% CI)	*p*

rs7620580					
AA	Null	129	176		
	Yes	69	52	**1.839 (1.201–2.816)**	**0.005**
AA + AG	Null	121	97	**1.714 (1.206–2.437)**	**0.003**
	Yes	64	37	0.760 (0.399–1.450)	0.406
rs2289843					
TT + TA	Null	197	220		
	Yes	97	68	1.601 (1.112–2.306)	0.011
AA	Null	53	53	1.080 (0.703–1.660)	0.725
	Yes	36	21	1.113 (0.523–2.368)	0.782
rs1708303					
AA	Null	152	193		
	Yes	88	67	**1.679 (1.145–2.461)**	**0.008**
AA + AG	Null	98	80	**1.534 (1.065–2.210)**	**0.022**
	Yes	45	22	1.015 (0.503–2.051)	0.966

Genotype	Smoking	IS (*n*)	Control (*n*)	OR (95% CI)	*p*

rs7620580					
AA	Null	117	117		
	Yes	82	111	0.739 (0.503–1.084)	0.122
AA + AG	Null	112	71	**1.577 (1.065–2.336)**	**0.023**
	Yes	74	63	1.008 (0.559–1.819)	0.979
rs2289843					
TT + TA	Null	177	159		
	Yes	119	129	1.062 (0.612–1.842)	0.832
AA	Null	52	29	1.486 (0.972–2.272)	0.068
	Yes	37	45	0.599 (0.313–1.145)	0.121
rs1708303					
AA	Null	145	133		
	Yes	96	127	**0.693 (0.486–0.988)**	**0.043**
AA + AG	Null	84	55	1.401 (0.926–2.118)	0.11
	Yes	60	47	1.206 (0.647–2.246)	0.556

Genotype	Drinking	IS (*n*)	Control (*n*)	OR (95% CI)	*p*

rs7620580					
AA	Null	146	153		
	Yes	53	75	0.741 (0.487–1.125)	0.159
AA + AG	Null	144	86	**1.755 (1.236–2.491)**	**0.002**
	Yes	42	48	0.706 (0.370–1.347)	0.291
rs2289843					
TT + TA	Null	224	194		
	Yes	72	94	0.777 (0.424–1.425)	0.415
AA	Null	66	45	1.286 (0.879–1.880)	0.195
	Yes	23	29	0.757 (0.371–1.542)	0.443
rs1708303					
AA	Null	182	168		
	yes	59	92	**0.592 (0.401–0.873)**	**0.008**
AA + AG	null	108	71	1.404 (0.974–2.024)	0.069
	yes	36	31	1.29 (0.649–2.562)	0.468

**Table 6:** Analysis of kalirin genotypic association with IS in subgroups.

*rs7620580*				

Gender				

Genotype	Woman		Man	
	OR (95% CI)	OR (95% CI)^*∗*^	OR (95% CI)	OR (95% CI)^*∗∗*^

AG	1.368 (0.866–2.162)	**1.988 (1.035–3.816)** ^▲^	**1.723 (1.154–2.572)** ^▲▲^	**1.776 (1.096–2.877)**
GG	1.583 (0.433–5.796)	2.236 (0.410–12.201)	2.135 (0.864–5.277)	**3.661 (1.215–11.036)**
Recessive	1.397 (0.387–5.048)	1.682 (0.319–8.876)	1.716 (0.704–4.179)	2.895 (0.982–8.541)
Additive	1.334 (0.896–1.987)	**1.804 (1.032–3.152)** ^▲^	**1.608 (1.158–2.233)** ^▲▲^	**1.832 (1.235–2.719)** ^▲▲^
Dominant	1.383 (0.884–2.163)	**2.007 (1.062–3.793)** ^▲^	**1.766 (1.200–2.600)** ^▲▲^	**1.923 (1.206–3.067)** ^▲▲^

BMI				

Genotype	BMI < 25 kg/m^2^		BMI ≥ 25 kg/m^2^	
	OR (95% CI)	OR (95% CI)^*∗∗∗*^	OR (95% CI)	OR (95% CI)^*∗∗∗∗*^

AA	1	1	1	1
AG	**1.658 (1.157–2.377)** ^▲▲^	1.598 (0.999–2.557)	1.302 (0.744–2.278)	1.966 (0.887–4.357)
GG	2.200 (0.866–5.462)	3.055 (0.918–10.166)	1.319 (0.367–4.744)	4.130 (0.858–19.874)
Recessive	1.802 (0.734–4.422)	2.565 (0.781–8.424)	1.181 (0.335–4.157)	3.105 (0.681–14.156)
Additive	**1.593 (1.175–2.160)** ^▲▲^	**1.651 (1.112–2.451)** ^▲^	1.236 (0.780–1.959)	**1.999 (1.077–3.711)** ^▲^
Dominant	**1.703 (1.200–2.416)** ^▲▲^	**1.699 (1.077–2.680)** ^▲^	1.304 (0.758–2.241)	**2.188 (1.019–4.700)** ^▲^

*rs2289843*				

Gender				

Genotype	Woman		Man	
	OR (95% CI)	OR (95% CI)^*∗*^	OR (95% CI)	OR (95% CI)^*∗∗*^

TT	1	1	1	1
TA	1.070 (0.643–1.779)	1.443 (0.687–3.033)	1.203 (0.763–1.895)	1.421 (0.825–2.446)
AA	1.635 (0.842–3.175)	**2.782 (1.134–6.826)** ^▲^	1.113 (0.656–1.891)	1.208 (0.645–2.264)
Recessive	1.565 (0.879–2.787)	**2.186 (1.035–4.620)** ^▲^	0.989 (0.636–1.539)	0.970 (0.573–1.642)
Additive	1.250 (0.903–1.730)	**1.658 (1.059–2.595)** ^▲^	1.059 (0.813–1.380)	1.108 (0.810–1.515)
Dominant	1.192 (0.734–1.936)	1.765 (0.872–3.570)	1.172 (0.765–1.796)	1.344 (0.810–2.231)

BMI				

Genotype	BMI < 25kg/m^2^		BMI ≥ 25 kg/m^2^	
	OR (95% CI)	OR (95% CI)^*∗∗∗*^	OR (95% CI)	OR (95% CI)^*∗∗∗∗*^

TT	1	1	1	1
TA	1.033 (0.692–1.541)	1.134 (0.678–1.897)	1.490 (0.782–2.839)	1.889 (0.733–4.866)
AA	1.129 (0.686–1.858)	1.370 (0.723–2.596)	1.548 (0.737–3.252)	1.569 (0.581–4.241)
Recessive	1.106 (0.722–1.693)	1.266 (0.730–2.197)	1.202 (0.646–2.236)	1.071 (0.474–2.420)
Additive	1.060 (0.827–1.357)	1.167 (0.850–1.602)	1.250 (0.860–1.817)	1.238 (0.754–2.034)
Dominant	1.059 (0.724–1.548)	1.197 (0.736–1.947)	1.510 (0.818–2.756)	1.741 (0.740–4.097)

*rs1708303*				

Gender				

Genotype	Woman		Man	
	OR (95% CI)	OR (95% CI)^*∗*^	OR (95% CI)	OR (95% CI)^*∗∗*^

AA	1	1	1	1
AG	1.613 (0.996–2.612)	1.663 (0.846–3.272)	1.444 (0.947–2.202)	1.497 (0.901–2.487)
GG	2.204 (0.644–7.548)	1.191 (0.236–6.015)	1.063 (0.261–4.330)	0.875 (0.168–4.564)
Recessive	1.886 (0.556–6.394)	1.020 (0.205–5.072)	0.953 (0.235–3.863)	0.778 (0.150–4.034)
Additive	**1.566 (1.045–2.345)** ^▲^	1.414 (0.809–2.470)	1.332 (0.914–1,941)	1.331 (0.848–2.090)
Dominant	**1.665 (1.046–2.652)** ^▲^	1.602 (0.837–3.068)	1.417 (0.938–2.141)	1.447 (0.881–2.374)

BMI				

Genotype	BMI < 25 kg/m^2^		BMI ≥ 25 kg/m^2^	
	OR (95% CI)	OR (95% CI)^*∗∗∗*^	OR (95% CI)	OR (95% CI)^*∗∗∗∗*^

AA	1	1	1	1
AG	**1.590 (1.094–2.309)** ^▲^	1.368 (0.841–2.227)	1.450 (0.783–2.686)	**2.435 (1.003–5.912)** ^▲^
GG	1.261 (0.433–3.674)	0.969 (0.248–3.781)	3.807 (0.434–33.355)	2.035 (0.164–25.291)
Recessive	1.086 (0.375–3.140)	0.880 (0.227–3.417)	3.437 (0.395–29.930)	1.651 (0.136–20.089)
Additive	**1.431 (1.036–1.978)** ^▲^	1.232 (0.813–1.867)	1.564 (0.912–2.683)	2.095 (0.962–4.562)
Dominant	**1.561 (1.086–2.244)** ^▲^	1.329 (0.829–2.131)	1.557 (0.854–2.840)	**2.397 (1.018–5.644)** ^▲^

^*∗*^adjusted for age, SBP, and drinking history in men;  ^*∗∗*^adjusted for age, SBP, LDL-C, and smoking history in women;  ^*∗∗∗*^adjusted for age, SBP, LDL-C, and smoking history in BMI < 25 kg/m2;  ^*∗∗∗∗*^adjusted for age, SBP, LDL-C, and smoking history in BMI ≥ 25 kg/m2; ^▲^*p* < 0.05, ^▲▲^*p* < 0.01.

**Table 7:** Haplotype analysis in cases and controls.

Haplotype	Cases (freq.)	Controls (freq.)	OR (95% CI)	*p*
A-T-A	202 (0.262)	281 (0.388)	**0.557 (0.446~0.695)**	**<0.001**
A-T-G	92 (0.120)	44 (0.060)	**2.138 (1.467~3.115)**	**<0.001**
A-A-A	242 (0.315)	219 (0.303)	1.064 (0.852~1.328)	0.585
A-A-G	28 (0.036)	35 (0.048)	0.745 (0.447~1.242)	0.257
G-T-A	84 (0.110)	50 (0.069)	**1.661 (1.152~2.395)**	**0.006**
G-A-A	85 (0.111)	64 (0.088)	1.295 (0.919~1.823)	0.139

All haplotypes with a frequency of < 0.03 were ignored for this analysis.

## References

[1] Meschia J. F., Bushnell C., Boden-Albala B. (2014). Guidelines for the primary prevention of stroke: A statement for healthcare professionals from the American heart association/American stroke association. *Stroke*.

[2] Murray C. J. L., Lopez A. D. (1997). Alternative projections of mortality and disability by cause 1990–2020: global burden of disease study. *The Lancet*.

[3] He J., Gu D., Wu X. (2005). Major causes of death among men and women in China. *The New England Journal of Medicine*.

[4] Markus H. S., Bevan S. (2014). Mechanisms and treatment of ischaemic stroke - Insights from genetic associations. *Nature Reviews Neurology*.

[5] Floßmann E., Schulz U. G. R., Rothwell P. M. (2004). Systematic Review of Methods and Results of Studies of the Genetic Epidemiology of Ischemic Stroke. *Stroke*.

[6] Bak S., Gaist D., Sindrup S. H., Skytthe A., Christensen K. (2002). Genetic liability in stroke: a long-term follow-up study of Danish twins. *Stroke*.

[7] O'Donnell M. J., Chin S. L., Rangarajan S. (2016). Global and regional effects of potentially modifiable risk factors associated with acute stroke in 32 countries (INTERSTROKE): a case-control study. *The Lancet*.

[8] Waters M. F., Hoh B. L., Lynn M. J. (2016). Factors associated with recurrent ischemic stroke in the medical group of the SAMMPRIS Trial. *JAMA Neurology*.

[9] Mandela P., Ma X.-M. (2012). Kalirin, a key player in synapse formation, is implicated in human diseases. *Neural Plasticity*.

[10] Cahill M. E., Xie Z., Day M. Kalirin regulates cortical spine morphogenesis and disease-related behavioral phenotypes.

[11] Tsai Y.-C., Riess O., Soehn A. S., Nguyen H. P. (2012). The Guanine Nucleotide Exchange Factor Kalirin-7 Is a Novel Synphilin-1 Interacting Protein and Modifies Synphilin-1 Aggregate Transport and Formation. *PLoS ONE*.

[12] Wu J.-H., Fanaroff A. C., Sharma K. C. (2013). Kalirin promotes neointimal hyperplasia by activating rac in smooth muscle cells. *Arteriosclerosis, Thrombosis, and Vascular Biology*.

[13] Boroumand M., Ziaee S., Zarghami N. (2014). The Kalirin gene rs9289231 polymorphism as a novel predisposing marker for coronary artery disease. *Laboratory Medicine*.

[14] Horne B. D., Hauser E. R., Wang L. (2009). Validation study of genetic associations with coronary artery disease on chromosome 3q13-21 and potential effect modification by smoking. *Annals of Human Genetics*.

[15] Wang L., Hauser E. R., Shah S. H. (2007). Peakwide mapping on chromosome 3q13 identifies the kalirin gene as a novel candidate gene for coronary artery disease. *American Journal of Human Genetics*.

[16] Ward-Caviness C., Haynes C., Blach C. (2013). Gene-smoking interactions in multiple Rho-GTPase pathway genes in an early-onset coronary artery disease cohort. *Human Genetics*.

[17] Zheng L., Sun Z., Li J. (2008). Pulse pressure and mean arterial pressure in relation to ischemic stroke among patients with uncontrolled hypertension in rural areas of China. *Stroke*.

[18] Yi P., Chen Z., YanZhao (2009). PCR/LDR/capillary electrophoresis for detection of single-nucleotide differences between fetal and maternal DNA in maternal plasma. *Prenatal Diagnosis*.

[19] Thomas G., Sinville R., Sutton S. (2004). Capillary and microelectrophoretic separations of ligase detection reaction products produced from low-abundant point mutations in genomic DNA. *Electrophoresis*.

[20] Shi Y., Li Z., Xu Q. (2011). Common variants on 8p12 and 1q24.2 confer risk of schizophrenia. *Nature Genetics*.

[21] Shi Y. Y., He L. (2005). SHEsis, a powerful software platform for analyses of linkage disequilibrium, haplotype construction, and genetic association at polymorphism loci. *Cell Research*.

[22] Li Z., Zhang Z., He Z. (2009). A partition-ligation-combination-subdivision em algorithm for haplotype inference with multiallelic markers: update of the SHEsis (http://analysis.bio-x.cn). *Cell Research*.

[23] Xin X.-Y., Song Y.-Y., Ma J.-F. (2009). Gene polymorphisms and risk of adult early-onset ischemic stroke: A meta-analysis. *Thrombosis Research*.

[24] Lin H.-F., Liao Y.-C., Liou C.-W., Liu C.-K., Juo S.-H. H. (2007). The phosphodiesterase 4D gene for early onset ischemic stroke among normotensive patients [13]. *Journal of Thrombosis and Haemostasis*.

[25] Olsson S., Jood K., Blomstrand C., Jern C. (2011). Genetic variation on chromosome 9p21 shows association with the ischaemic stroke subtype large-vessel disease in a Swedish sample aged </= 70. *European journal of neurology*.

[26] Ferraro F., Ma X.-M., Sobota J. A., Eipper B. A., Mains R. E. (2007). Kalirin/Trio Rho guanine nucleotide exchange factors regulate a novel step in secretory granule maturation. *Molecular Biology of the Cell*.

[27] Rabiner C. A., Mains R. E., Eipper B. A. (2005). Kalirin: A dual Rho guanine nucleotide exchange factor that is so much more than the sum of its many parts. *Neuroscientist*.

[28] Ma X.-M., Kiraly D. D., Gaier E. D. (2008). Kalirin-7 is required for synaptic structure and function. *Journal of Neuroscience*.

[29] Penzes P., Johnson R. C., Sattler R. (2001). The neuronal Rho-GEF Kalirin-7 interacts with PDZ domain-containing proteins and regulates dendritic morphogenesis. *Neuron*.

[30] Ma X.-M., Huang J.-P., Kim E.-J. (2011). Kalirin-7, an important component of excitatory synapses, is regulated by estradiol in hippocampal neurons. *Hippocampus*.

[31] Ma X. M., Huang J., Wang Y., Eipper B. A., Mains R. E. (2003). Kalirin, a multifunctional Rho guanine nucleotide exchange factor, is necessary for maintenance of hippocampal pyramidal neuron dendrites and dendritic spines. *The Journal of Neuroscience*.

[32] Ma X.-M., Wang Y., Ferraro F., Mains R. E., Eipper B. A. (2008). Kalirin-7 is an essential component of both shaft and spine excitatory synapses in hippocampal interneurons. *Journal of Neuroscience*.

[33] Xie Z., Srivastava D. P., Photowala H. (2007). Kalirin-7 controls activity-dependent structural and functional plasticity of dendritic spines. *Neuron*.

[34] Beresewicz M., Kowalczyk J. E., Zabłocka B. (2008). Kalirin-7, a protein enriched in postsynaptic density, is involved in ischemic signal transduction. *Neurochemical Research*.

[35] Ratovitski E. A., Alam M. R., Quick R. A. (1999). Kalirin inhibition of inducible nitric-oxide synthase. *Journal of Biological Chemistry*.

[36] Boucher P., Gotthardt M., Li W.-P., Anderson R. G. W., Herz J. (2003). LRP: Role in vascular wall integrity and protection from atherosclerosis. *Science*.

[37] Wu J.-H., Zhang L., Fanaroff A. C. (2012). G protein-coupled receptor kinase-5 attenuates atherosclerosis by regulating receptor tyrosine kinases and 7-transmembrane receptors. *Arteriosclerosis, Thrombosis, and Vascular Biology*.

[38] Subramanian V., Golledge J., Ijaz T., Bruemmer D., Daugherty A. (2010). Pioglitazone-induced reductions in atherosclerosis occur via smooth muscle cell-specific interaction with PPAR*γ*. *Circulation Research*.

[39] Nohria A., Grunert M. E., Rikitake Y. (2006). Rho kinase inhibition improves endothelial function in human subjects with coronary artery disease. *Circulation Research*.

[40] Bradshaw N. J., Porteous D. J. (2012). DISC1-binding proteins in neural development, signalling and schizophrenia. *Neuropharmacology*.

[41] Hill J. J., Hashimoto T., Lewis D. A. (2006). Molecular mechanisms contributing to dendritic spine alterations in the prefrontal cortex of subjects with schizophrenia. *Molecular Psychiatry*.

[42] Lesch K.-P., Timmesfeld N., Renner T. J. (2008). Molecular genetics of adult ADHD: Converging evidence from genome-wide association and extended pedigree linkage studies. *Journal of Neural Transmission*.

[43] Dang M., Wang Z., Zhang R. (2015). KALRN Rare and Common Variants and Susceptibility to Ischemic Stroke in Chinese Han Population. *NeuroMolecular Medicine*.

[44] Krug T., Manso H., Gouveia L. (2010). Kalirin: A novel genetic risk factor for ischemic stroke. *Human Genetics*.

[45] Olsson S., Jood K., Melander O. (2011). Lack of association between genetic variations in the KALRN region and ischemic stroke. *Clinical Biochemistry*.

